# Risk Factors for Adverse Maternal and Fetal Outcomes in Women With Confirmed aPL Positivity: Results From a Multicenter Study of 283 Pregnancies

**DOI:** 10.3389/fimmu.2018.00864

**Published:** 2018-05-07

**Authors:** Micaela Fredi, Laura Andreoli, Elena Aggogeri, Elisa Bettiga, Maria Grazia Lazzaroni, Véronique Le Guern, Andrea Lojacono, Nathalie Morel, Jean Charles Piette, Sonia Zatti, Nathalie Costedoat-Chalumeau, Angela Tincani

**Affiliations:** ^1^Rheumatology and Clinical Immunology Unit, ASST Spedali Civili di Brescia, Brescia, Italy; ^2^Department of Clinical and Experimental Sciences, University of Brescia, Brescia, Italy; ^3^Internal Medicine Department, Cochin Hospital, Paris Descartes University, Paris, France; ^4^Obstetrics and Gynecology Department, ASST Spedali Civili di Brescia and University of Brescia, Brescia, Italy; ^5^Centre de référence maladies auto-immunes et systémiques rares d’Ile de France, Internal Medicine Department, Pitié-Salpêtrière Hospital, Assistance Publique Hopitaux De Paris (AP-HP), Paris, France; ^6^Université Pierre et Marie Curie, Paris, France; ^7^Centre de référence maladies auto-immunes et systémiques rares d’Ile de France, Internal Medicine Department, Cochin Hospital, Assistance Publique Hopitaux De Paris (AP-HP), Paris, France; ^8^Université Paris Descartes-Sorbonne Paris Cité, Paris, France; ^9^INSERM U 1153, Center for Epidemiology and Statistics Sorbonne Paris Cité (CRESS), Paris, France

**Keywords:** pregnancy, adverse pregnancy outcome, antiphospholipid antibodies, autoimmune thyroiditis, risk factors, therapy

## Abstract

**Objective:**

Antiphospholipid antibodies positivity (aPL) is considered as a risk factor for adverse pregnancy outcome (APO). The aim of this study was to determine the risk factors for APO in patients with confirmed aPL positivity, isolated (aPL carriers) or associated with a definite primary antiphospholipid syndrome (PAPS).

**Methods:**

The clinical and laboratory features of 283 pregnancies occurring between 2000 and 2014 in 200 women were collected in three institutions.

**Results:**

The rate of live birth was 87.9% and APO was observed in 50 cases (17.7%). Multivariate analysis showed that the independent variables related to APO were the concomitant diagnosis of an organ-specific autoimmune disease (*p* = 0.012, odds ratio (OR) 3.29, confidence interval (CI) 95% 1.29–8.38) and the presence of low complement levels during the first trimester (*p* = 0.02, OR 2.3, CI 95% 1.17–9.15). No statistical differences were found in APO occurrence among patients treated with low-dose aspirin (LDA) versus those treated with LDA plus heparin (LMWH), but LDA + LMWH was more frequently administered in patients with triple aPL positivity (*p* = 0.001, OR 3.21, CI 95% 1.48–7.11) and with PAPS (*p* < 0.001, OR 8.08, CI 95% 4.3–15.4). Based on clinical history, the patients were divided into four groups: obstetric, thrombotic, non-criteria antiphospholipid syndrome (clinical non-criteria), and aPL carriers. APOs were more frequent in the thrombotic group (24%). Seven patients had a thrombotic event during pregnancy or puerperium (2.4%).

**Conclusion:**

Maternal and fetal complications were observed in some aPL-positive patients despite their efficient management according to the current recommendations. A higher risk of APO was observed in patients with a previous thrombosis and/or more complex autoimmune phenotype.

## Introduction

The antiphospholipid syndrome (APS) is an acquired systemic autoimmune disease characterized by the presence of obstetrical morbidity and recurrent thrombotic vascular events associated with antiphospholipid antibodies (aPL). aPL is a heterogeneous group of autoantibodies reacting against phospholipids, phospholipid–protein complexes, and phospholipid-binding proteins (1). The clinical classification “criteria” include arterial/venous thrombosis and obstetric morbidity (more than three consecutive early pregnancy loss, fetal death (FD) at or beyond 10 week of gestation, and early severe preeclampsia or placental insufficiency necessitating delivery before 34 weeks of gestation). The laboratory criteria include the persistent positivity for at least one test among lupus anticoagulant (LA), anticardiolipin (aCL), and anti beta2glycoprotein I antibodies (anti-B2GPI). According to the criteria, both aCL IgG/IgM or/and anti-B2GPI IgG/IgM should be at medium or high titer.

Furthermore, aPL may also be associated with less specific clinical features, defined as “non-criteria”(1). These include heart valve disease, livedo reticularis, thrombocytopenia, aPL nephropathy, neurological manifestations such as epilepsy and cognitive dysfunction as well as previous pregnancy morbidity which do not fulfill the formal “criteria” for APS (two consecutive early pregnancy losses, late-onset preeclampsia, etc.). The presence of aPL antibodies has also been detected in “aPL carriers,” subjects without any clinical features of APS (with or without systemic autoimmune diseases). Primary APS was defined as the absence of associated systemic connective tissue disease (CTD).

The clinical management of pregnant patients with aPL aims at preventing obstetric complications and maternal thrombotic events. Combination therapy of low-dose aspirin (LDA) and heparin is regarded as conventional treatment for patients with an established diagnosis of obstetric APS (2, 3), generally resulting in over 70% successful pregnancies. However, a significant number of pregnancies are still complicated or unsuccessful in women with APS. In patients not fulfilling the criteria for definite APS, the management is still debated and different protocols are applied during pregnancy with contrasting results. In clinical practice, LDA is usually administered to patients with aPL (4). However, a recent systematic review, including three studies of aPL-positive patients not fulfilling the clinical criteria for APS (154 pregnancies), did not find clear evidence of LDA superiority in the prevention of pregnancy loss and complications (5). The discrepancy between the published literature and the “real life” emphasizes the need to better classify the patients according to the stratification of obstetric risk. In fact, the definition of the risk factors for pregnancy failure will provide an objective tool for tailoring the management of patients on their individual risk profile. Therefore, the aim of this collaborative work was to assess the risk factor of obstetric complications in patients with confirmed aPL positivity with or without a diagnosis of primary antiphospholipid syndrome.

## Patients and Methods

### Study Cohort

Medical records of pregnant women with confirmed positivity for aPL antibodies attending three referral centers (Rheumatology or Internal Medicine Departments with consolidate experience on APS) from January 2000 to December 2014 were retrospectively evaluated. Patients with a diagnosis of systemic CTD (according to the international classification criteria) at the beginning of the follow-up were excluded. The presence of other autoantibodies and/or low complement levels was not considered an exclusion criterion if not associated with clinical manifestations specific for CTDs.

This study was performed according to the principles of the Declaration of Helsinki with written informed consent from all subjects and was approved by the Ethic Committee of the Promoting Centre (approval number 1088) and it has been approved by the other centers.

### Autoantibodies Detection

Lupus anticoagulant was detected by coagulation assay according to the guidelines of the International Society on Thrombosis and Haemostasis (6), while aCL and anti-B2GPI IgG and IgM by ELISA according to the current recommendations (7). Antinuclear antibodies (ANA), anti-double-stranded DNA (anti-dsDNA) antibodies, antibodies to extractable nuclear antigens (anti-ENA), and complement C3 and C4 fractions were detected as for clinical practice. Antiphospholipid antibodies were considered positive if confirmed at least 12 weeks apart, according to the classification “criteria.” In each center, the tests were performed in a referral laboratory certified for diagnosis. Data concerning the prevalence of other antiphospholipid antibodies not included in the classification criteria were not collected because they were not routinely performed.

### Adverse Pregnancy Outcome (APO) Definition

In this study, we considered the following events as aPL-related APO: spontaneous abortions (SAs) (<10 weeks of gestation), FD (≥10 weeks of gestation), neonatal death before hospital discharge due to complications of prematurity, preterm delivery before 34 weeks of gestation with or without preeclampsia, hemolysis, elevated liver enzymes, and low platelet (HELLP) syndrome (concomitant presence of thrombocytopenia, evidence of hepatic dysfunction, and hemolysis), and small for gestational age babies (SGA) associated with abnormal Doppler flow velocimetry. Pregnancies with an identifiable other cause for APO (i.e., anatomical abnormalities, cervix dilatation) were excluded from statistical analysis.

### Statistical Evaluation

Categorical variables were reported as proportion and/or percentage, continuous variables as mean (±SD) values. Fisher’s exact test or chi-squared test for categorical variables and Student’s *t*-test or Wilcoxon–Mann–Whitney test for continuous variables were applied as appropriate. For the multivariate analyses, we included the features associated to APO at the univariate analysis. Multivariate analysis was conducted by logistical regression model (Statview). *P*-values of <0.05 were considered significant and Odds Ratio (OR) with 95% confidence interval (95% CI) was indicated.

## Results

### Patients

The 200 patients included were Caucasian (*n* = 177, 88.5%), African (*n* = 8, 4%), and others (*n* = 15, 7.5%). An organ-specific autoimmune disease was diagnosed in 28 women: autoimmune thyroiditis (*n* = 20, 10%), celiac disease (*n* = 2, 1%), cutaneous psoriasis (*n* = 2, 1%), autoimmune hepatitis (*n* = 2, 1%), autoimmune thyroiditis and celiac disease (*n* = 1, 0.5%), and primary biliary cirrhosis (*n* = 1, 0.5%).

Sixty patients (30%) had at least one modifiable cardiovascular risk factor (cigarette smoke, obesity, arterial hypertension) at pregnancy onset. Inherited thrombophilic factors (factor II and factor V mutation, protein C/protein S/antithrombin deficiency) were available for 168 pregnancies and abnormalities were found in 16 patients (9.5%).

According to the classification “criteria” for APS, the patients had obstetric morbidity only (O-APS; *n* = 85, 42.5%), thrombotic events (with or without pregnancy morbidity) (T-APS; *n* = 42, 21%), clinical “non-criteria” manifestations (NC-APS; *n* = 39, 19.5%) or aPL positivity without any clinical manifestations (aPL carriers; *n* = 34, 17%). The details about the clinical criteria manifestations at study onset are reported in Table 1. One hundred and fifty patients had 344 previous pregnancies not followed in one of the three centers, with a live birth in 98 cases (28%).

**Table 1:** Clinical criteria manifestations at study onset.^a^

Clinical manifestation patients with primary antiphospholipid syndrome (APS)	

Arterial/venous thrombotic manifestation^a^	Number of patients 42 (%)
Deep venous thrombosis	32 (76.2)
Pulmonary embolism	8 (19.4)
Stroke	7 (16.6)
Myocardial infarction	3 (7.1)
Peripheral arterial thrombosis	2 (4.8)
Gastrointestinal venous tract thrombosis	1 (2.3)

**Obstetrical manifestation^a^**	**Number of patients 99 (%)^b^**

Fetal deaths (≥1 event)	57 (57.5)
Premature births before 34 weeks	20 (20.2)
Spontaneous abortion (SA) (≥3 consecutive events)	17 (17.2)
Preeclampsia^c^/Eclampsia^d^/Hemolysis, elevated liver enzymes, and low platelets (HELLP syndrome)^e^	16 (16.2)

**Clinical manifestation of “non-criteria APS” patients 39 (%)**	

Obstetrical non-criteria (i.e., <3 consecutive or not consecutive SA)	28 (71.8)
Premature births >34 weeks but <37 weeks	20 (51.3)
Preeclampsia/Eclampsia/HELLP syndrome/IUGR^f^ after 34 weeks	5 (12.8)
Livedo reticularis	5 (12.8)
Thrombocytopenia	3 (7.7)
Chorea	2 (5.2)
Valvulopathy	2 (5.2)
Hemolytic anemia	1 (2.6)

*^a^The same patient can be included in more than one category. The obstetrical manifestations were previously described (see Patients and Methods)*.

*^b^We considered the patients included in the O-APS category (85 patients) and 14 patients in the T-APS who also pregnancy morbidity*.

*^c^Preeclampsia, increased blood pressure associated with proteinuria in pregnancy; proteinuria is defined as the excretion of 300 mg of protein or greater in a 24-h specimen*.

*^d^Eclampsia, onset of seizures during preeclampsia*.

*^e^HELLP syndrome, concomitant presence of severe thrombocytopenia (platelets ≤50,000/μl), evidence of hepatic dysfunction (liver enzymes ≥70 IU/l), and evidence suggestive of hemolysis (total serum lactate dehydrogenase ≥600 IU/l)*.

*^f^IUGR, intrauterine growth restriction (assessed by ultrasound as a fetal abdominal circumference in the <5th percentile)*.

### Frequency of positive autoantibodies

The results of the three aPL assays were available for all the patients. LA was detectable in 80 patients (40%). aCL antibodies were positive in 131 patients (65.5%), 101 IgG and 60 IgM (50.5 and 30%); anti-B2GPI in 124 (62%), 84 IgG and 71 IgM (42 and 35.5%). A triple aPL positivity was observed in 46 women (23%) while double in 43 (21.5%) and single in 111 (55.5%). ANAs were persistently positive in 75 patients (37.5%), anti-dsDNA in 9 (4.5%), and anti-ENA in 8 (4%) (anti-Ro/SSA in 6 cases, anti-Sm/RNP in 2). Complement levels were tested at the beginning of the pregnancy in 134 patients and were found to be low in 16 (12%).

### Pregnancy Outcome

During the study period, data of 283 pregnancies were collected. The mean maternal age at conception was 32.4 ± 5.1 years. The outcome of the pregnancies was as follows: 248 live births (88%) with 251 babies (3 twin pregnancies) at a mean gestational age of 37.6 ± 3.4 weeks (range 25.6–41.5), SA before the 10 weeks of gestation (*n* = 20), FD (*n* = 12), voluntary (*n* = 1), and medical terminations (*n* = 2: one Steinert’s syndrome and one trisomy 18).

At least one complication occurred in 110 pregnancies (38.9%) (see Table 2) including the neonatal death of a child born at 27 weeks to a patient with HELLP syndrome.

**Table 2:** Outcome of 283 pregnancies in 200 patients during the follow-up.

Gestational outcome and obstetrical complications*	283 Pregnancies (%)
Spontaneous abortion	20 (7.1)
Fetal death	12 (4.2)
Voluntary/medically induced interruption of pregnancy	3 (1)
**Deliveries**	**248 (87.6)**
Live births	247 (87.3)
Neonatal death^a^	1 (0.3)
Preterm deliveries <37 weeks	41 (14.4)
Preterm deliveries <34 weeks	16 (5.7)
Preeclampsia	22 (7.8)
Small for gestational age (SGA)^b^	17 (6)
Intrauterine growth restriction	11 (3.9)
Preterm premature rupture of membranes^c^	7 (2.4)
HELLP syndrome^d^	7 (2.4)
Gestational diabetes	13 (4.6)
Gestational hypertension^e^	4 (1.4)

*The same patient can be included in more than one category.

*^a^Neonatal death, death of a formed fetus alive at birth in the first 28 days of life*.

*^b^SGA, small for gestational age was defined as a birth weight in the <10th percentile for gestational age*.

*^c^Preterm premature rupture of membranes was defined as rupture of the membranes before 37 weeks of gestation*.

*^d^HELLP syndrome concomitant presence of severe thrombocytopenia (platelets ≤50,000/μl), evidence of hepatic dysfunction (liver enzymes ≥70 IU/l), and evidence suggestive of hemolysis (total serum lactate dehydrogenase ≥600 IU/l)*.

*^e^Gestational hypertension, onset of hypertension after 20 weeks of gestation, without proteinuria*.

Fifty out of 110 (45.4%) complicated pregnancies were defined as APO (17.7% of all pregnancies) (Table 3). Tables 4 and 5 report the univariate statistical comparison of clinical and laboratory features between APO and uneventful pregnancies. In multivariate analysis, the independent features related to APO were either the concomitant diagnosis of an organ-specific autoimmune disease (*p* = 0.012, OR 3.29, CI 95% 1.29–8.38) or the presence of low complement levels at conception or during the first trimester (*p* = 0.02, OR 2.3, CI 95% 1.17–9.15). The positivity of other autoantibodies (i.e., ANA medium titer, anti-ENA, anti-dsDNA) almost reached the significant threshold (*p* = 0.06, OR 2.34, CI 95% 0.96–5.72).

**Table 3:** Lists of adverse pregnancy outcome (APO) related to aPL presence.

APO related to aPL*	Pregnancies (*n* = 50)
Spontaneous abortion	20 (40)
Fetal death	12 (24)
Neonatal death due to prematurity	1 (2)
Preterm deliveries <34 weeks	16 (23)
Small for gestational age (SGA) associated with abnormal Doppler flow velocimetry	4 (8)
Hemolysis, elevated liver enzymes, and low platelet	7 (14)

*The same patient can be included in more than one category.

**Table 4:** Univariate analysis of clinical features in successful and adverse pregnancy outcome (APO) pregnancies.

Clinical/serological features	APO (*n* = 50) (17.7%)	Successful pregnancies (*n* = 233) (82.3%)	*p*-Value	OR (CI 95%)
Age at the onset ≥35 years (*n* = 107)	20/50 (40)	87/233 (37.3)	NS	_
Formal diagnosis of antiphospholipid syndrome (APS) (*n* = 190)	38/50 (76)	152/233 (65.2)	NS	–
Organ-specific autoimmune disease^a^ (*n* = 38)	12/50 (24)	26/233 (11.2)	**0.016**^e^	**2.51 (1.09–5.75)**
Previous thrombosis (*n* = 66)	16/50 (32)	50/233 (21.5)	NS	–
Previous pregnancy morbidity (*n* = 151)	27/50 (54)	124/233 (53.2)	NS	–
Previous premature birth (*n* = 32)	10/50 (20)	22/233 (9.4)	**0.032**^f^	**2.39 (0.99–5.82)**
Previous ≥3 spontaneous abortion (*n* = 29)	4/50 (8)	25/233 (10.7)	NS	–
Prior pregnancy morbidity and thrombosis (*n* = 27)	5/50 (10)	22/233 (9.4)	NS	–
Other APS-related manifestations^b^ (*n* = 48)	16/50 (32)	32/233 (13.7)	**0.002**^f^	**2.95 (1.38–6.29)**
Acquired risk factors for thrombosis^c^ (*n* = 90)	21/50 (42)	69/233 (29.6)	NS	–
Inherited thrombophilia (data available for 237 pregnancies)^d^ (*n* = 23)	5/40 (12.5)	18/197 (9.1)	NS	–

*^a^Associated autoimmune organ disease, thyroiditis, autoimmune hepatitis, primary biliary cirrhosis, psoriasis, celiac disease*.

*^b^Other APS-related manifestations, thrombocytopenia, epilepsy, headache, livedo reticularis, heart valve lesions, and hemolytic anemia*.

*^c^Acquired risk factors for thrombosis, hypertension, BMI >30 kg/m^2^, and smoke*.

*^d^Inherited thrombophilia, factor II and factor V mutation, protein C and S and antithrombin III deficiency*.

*NS, not statistically significant*.

*^e^Significant at multivariate analysis, *p* = 0.012 (OR 3.29, CI 95% 1.29–8.38)*.

*^f^Not significant at multivariate analysis*.

**Table 5:** Univariate analysis of serological features in successful and adverse pregnancy outcome (APO) pregnancies.

Clinical/serological features	APO (*n* = 50) (17.7%)	Successful pregnancies (*n* = 233) (82.3%)	*p*-Value	OR (CI 95%)
Lupus anticoagulant (LA) positivity (*n* = 125)	29/50 (58)	96/233 (41.2)	**0.03**^c^	**1.97 (1.01–3.83)**
LA single positivity (*n* = 20)	2/50 (4)	18/233 (7)	NS	**–**
IgG anticardiolipin (aCL) (*n* = 153)	36/50 (72)	117/233 (50.2)	**0.005**^c^	**2.54 (1.25–5.26)**
IgG aCL single positivity (*n* = 37)	8/50 (16)	29/233 (12.4)	NS	
IgM aCL (*n* = 53)	13/50 (26)	40/233 (17.2)	NS	–
IgM aCL single positivity (*n* = 6)	1/50 (2)	5/233 (2.1)	NS	–
IgG anti-B2GPI (*n* = 134)	22/50 (44)	112/233 (48.1)	NS	–
IgG anti-B2GPI single positivity (*n* = 36)	4/50 (8)	32/233 (13.7)	NS	
IgM anti-β2GPI positivity (*n* = 107)	17/50 (34)	90/233 (38.6)	NS	–
IgM anti-B2GPI single positivity (*n* = 24)	2/50 (4)	22/233 (9.4)	NS	–
Single aPL positivity (*n* = 144)	20/50 (40)	124/233 (53.2)	NS	–
Double aPL positivity (*n* = 55)	8/50 (16)	47/233 (21)	NS	–
Triple aPL positivity (*n* = 84)	22/50 (44)	62/233 (26.6)	**0.015**^c^	**2.16 (1.10–4.25)**
Other autoantibodies^a^ (*n* = 78)	21/50 (42)	57/233 (24.5)	**0.012**^c^	**2.23 (1.12–4.42)**
Low complement levels^b^ (data available for 195 pregnancies) (*n* = 27)	11/34 (32.3)	16/161 (9.9)	**0.001**^d^	**4.33 (1.63–11.46)**

*^a^Other autoantibodies, antinuclear antibody ≥1:320, anti-double-stranded DNA, antibodies to extractable nuclear antigens*.

*^b^Low complement levels, decrease in C3 and/or C4 at conception or during first trimester*.

*NS, not statistically significant*.

*^c^Not significant at multivariate analysis*.

*^d^Significant at multivariate analysis, *p* = 0.02 (OR 2.3, CI 95% 1.17–9.15)*.

The most prevalent autoimmune disease was thyroiditis (22 patients with 31 pregnancies) which resulted in a significantly higher risk of SA (*p* = 0.014, OR 4.1; CI 95% 1.26–12.73). The only other association was found to be with low levels of C3 and/or C4 and fetal losses (*p* = 0.008, OR 9.56, CI 95% 1.66–56.4).

### Pregnancy Outcome in Patients With or Without a Formal Diagnosis of APS

During the follow-up, 137 patients classified as T-APS or O-APS had 190 pregnancies, whereas 73 I-APS or aPL carrier patients had 93. APO in these two groups was not significantly different (20 versus 13%; *p* = 0.19). The frequency distribution of APO in four subgroups (T-APS, O-APS, NC-APS, and aPL carrier) was 24.2, 18.7, 9.2, and 17.9%, respectively. In Tables 6 and 7, we describe the serological profile, the different APO, and the risk factor associated with APO in the four subgroups.

**Table 6:** Description of serological profile, treatment, and prevalence of adverse pregnancy outcome (APO) in the subgroups (all pregnancies included in the study).

	Serological profile			
	O-APS, *n* = 124 (%)	T-APS, *n* = 66 (%)	NC-APS, *n* = 54 (%)	aPL carrier, *n* = 39 (%)
Single aPL positivity	75 (60.4)	16 (24.2)	32 (59.2)	21(53.8)
Double aPL positivity	20 (16.1)	14 (21.2)	11(20.3)	10 (25.6)
Triple aPL positivity	29 (23.3)	36 (54.5)	11(20.3)	8 (20.6)

	**Treatment**			

LDA monotherapy	17 (13.7)	1 (1.5)	23 (42.6)	26 (66.6)
LMWH monotherapy	0 (0)	5 (7.5)	0 (0)	0 (0)
Combination treatment (LDA + LMWH) prophylactic dose	97 (78.2)	21 (31.2)	31 (57.4)	12 (30.8)
Combination treatment (LDA + LMWH) therapeutic dose	10 (8)	39 (59.1)	0 (0)	1 (2.6)
Hydroxychloroquine	8 (6.4)	19 (28.8)	3 (5.5)	2 (5.1)
Steroids	4 (3.2)	9 (13.6)	3 (5.5)	4 (10.2)

	**APO^a^**			
	**O-APS, *n* = 22 (17.7%)**	**T-APS, *n* = 16 (24.2%)**	**NC-APS, *n* = 5 (9.2%)**	**aPL carrier, *n* = 7 (17.9%)**

Spontaneous abortion	9 (41)	8 (50)	2 (40)	1 (14.2)
Fetal death	4 (18.2)	4 (25)	1 (20)	3 (42.8)
Neonatal death due to prematurity	0 (0)	0 (0)	0 (0)	1 (14.2)
Preterm deliveries <34 weeks	7 (31.8)	4 (25)	2 (40)	3 (42.8)
Small for gestational age associated with abnormal Doppler flow velocimetry	2 (9)	0 (0)	1(20)	1 (14.2)
Hemolysis, elevated liver enzymes, and low platelets	2 (9)	3 (18.7)	0 (0)	2 (28.5)

*LDA, low-dose aspirin; LMWH, low-molecular-weight heparin*.

*^a^The same patient can be included in more than one category*.

**Table 7:** Laboratory and clinical features associated with adverse pregnancy outcome (APO) in the four subgroups.

	APO *n*(%)	Features associated with APO	*p*-Value	OR (95% CI)
T-APS, 42 patients, 66 pregnancies	16 (24)	Prior venous thrombotic event	0.028	>20 (0.09–150)

O-APS, 85 patients, 124 pregnancies	22 (18)	Previous premature birth	0.037	2.85 (0.92–8.78)
		Other autoantibodies^a^	0.023	3.02 (1.01–9.02)
		Low complement levels^b^	0.04	3.63 (0.83–15.6)

NC-APS, 39 patients, 54 pregnancies	5 (9)	LA positive (any combination)	0.03	1.97 (1.01–3.83)
		Anti-B2GPI IgG positive (any combination)	0.017	6.91 (1.28–49)

aPL carrier, 34 patients, 39 pregnancies	7 (18)	Triple aPL-positive	0.022	9.33 (1.13–90.3)

*^a^Other autoantibodies, antinuclear antibody ≥1:320, anti-double-stranded DNA, antibodies to extractable nuclear antigens*.

*^b^Low complement levels, decrease in C3 and/or C4 at conception or during first trimester*.

### Therapy

All patients were treated with LDA (*n* = 278; 98.2%) and/or low-molecular-weight heparin (LMWH; *n* = 216; 76.3%). In 85% of the cases, LDA was used before the eighth week of gestation. LMWH was introduced before the eighth week of gestation in 164 pregnancies (76% of all pregnancies treated with heparin). Combination therapy was more frequent in patients with triple aPL positivity compared to single/double positivity (*p* = 0.001, OR 3.21, CI 95% 1.48–7.11). In addition, immunomodulatory or immunosuppressive therapy was recorded in 42 pregnancies with hydroxychloroquine and corticosteroids in 32 (11.3%) and 16 (5.7%) cases, respectively. Moreover, combination therapy was used more frequently in patients satisfying the criteria for primary APS compared to the others (*p* < 0.001, OR 8.086, CI 95% 4.3–15.4). Table 6 outlines the distribution of treatments in the four subgroups of patients. There were no significant differences in the rate of APO among the patients treated with LDA only or the combination therapy (9/47 in LDA versus 58/231 in LDA + LMWH) (Table 8). Moreover, the number of APO was neither related to the time of introduction of the combination therapy nor to the heparin dosage. Interestingly, three out of five pregnancies treated only with LMWH experienced an adverse outcome (*p* = 0.04, OR 7.37, CI 95% 0.96–65).

**Table 8:** Comparison of treatment in successful and complicated pregnancy.

Treatment	Adverse pregnancy outcome (*n* = 50) (17.7%)	Successful pregnancies (*n* = 233) (82.3%)	*p*-Value
LDA monotherapy (*n* = 67)	9/50 (18)	58/233 (24.9)	NS
LMWH monotherapy (*n* = 5)	3/50 (6)	2/233 (0.8)	0.040*
LDA + LMWH (*n* = 211)	38/50 (76)	173/233 (74.2)	NS
LDA + LMWH prophylactic dosage (*n* = 161)	28/50 (56)	133/233 (57.1)	NS
LDA + LMWH therapeutic dosage (*n* = 50)	10/50 (20)	40/233 (17.2)	NS
LDA + LMWH, start at positive pregnancy test (*n* = 143)	28/50 (56)	115/233 (49.3)	NS
LDA + LMWH, start between 6 and 8 weeks of gestation (*n* = 16)	0/50 (0)	16/233 (6.8)	NS
LDA + LMWH, Start between 9 and 12 weeks of gestation (*n* = 22)	6/50 (12)	16/233 (6.8)	NS
LDA + LMWH, start after 12 weeks of gestation (*n* = 30)	4/50 (8)	26/233 (11.1)	NS
Hydroxychloroquine (*n* = 32)	6/50 (12)	26/233 (11.2)	NS
Steroids (*n* = 16)	4/50 (8)	12/233 (5.1)	NS

*LDA, low-dose aspirin; LMWH, low-molecular-weight heparin*.

*OR 7.37; CI 95% 0.96–65.

### Maternal Outcome

Seven patients (2.5%) experienced a thrombotic event during pregnancies or puerperium: three during pregnancies (all during the first trimester) and four during puerperium (in three cases within 1 week after delivery and in one case 1 month after birth). No patient received ovulation induction therapy. Of these thrombotic events, four were venous (three deep venous thrombosis and one pulmonary embolism) and three arterial [one myocardial infarction and two catastrophic antiphospholipid syndrome (CAPS)]. Five of these events occurred in the T-APS subgroup, one in O-APS, and one in aPL carrier. In addition, the serological profile of these patients showed that four (53%) were triple positive, one double positive (LA and aCL), and two single positive (aCL). Six out of these seven patients (85%) were already receiving antithrombotic prophylaxis at the time of the event. The two patients who experienced CAPS have been previously described (8).

## Discussion

The aim of the present study was to analyze the gestational and maternal outcome in patients with confirmed positivity for antiphospholipid antibodies (aPL) followed up during their pregnancies. The most important exclusion criteria were the concomitant presence of another defined CTD, primarily SLE, a condition recognized as an independent risk factor for pregnancy failure in previous multicenter studies (9, 10).

The gestational outcome of these pregnancies significantly improved as compared to historic ones, with a live birth rate of 87.9%. In fact, a collaborative European study (EUROAPS) reported a live birth rate of 77.7% in patients with pure obstetric APS (11), while a recent retrospective Italian Study (PREGNANT) (12) reported 54.3% in patients with primary APS. Our results are difficult to compare with previously published cohorts as they mainly analyzed pregnancy outcome in patients with established APS but also with the incomplete form only.

Despite the high rate of live births in our study, maternal–fetal complications still occurred, and APOs related to aPL were identified in 17.7% of the pregnancies. Several studies have previously attempted to identify the clinical and laboratory variables predictive of APO (9, 10), and a concomitant SLE diagnosis and prior thrombotic events were found to be associated with poor pregnancy outcome. Moreover, previous works have identified the triple aPL positivity as an independent risk factor (9), while a prospective multicenter study (13, 14) supported the key role of LA as the main predictor of APO.

In this study, we show by means of multivariate analysis that the presence of a concomitant organ-specific autoimmune disease and/or low levels of C3 and/or C4 at conception are the only two independent factors associated with APO, although it seems to be associated with several clinical and serological factors from the univariate analysis. Autoimmune thyroiditis accounted for 71% of organ-specific autoimmune disease in our cohort and was associated with SA as previously reported (15). Particularly, we did not find any association between APO and any peculiar aPL positivity. This may be due to the small number of complicated pregnancies collected and/or to the fact that patients with LA or triple positivity were more frequently treated with the combination therapy. This observation could also account for the lack of difference in pregnancy outcome between women treated with combination therapy and those receiving LDA alone, which is generally considered as a less effective treatment (2).

Low complement levels at the beginning of pregnancy were also observed as an independent risk factor for APO. The role of complement activation in the pathogenesis of APS pregnancy morbidity is an intriguing question. Previous studies have demonstrated that the activation of complement components C3, C4, and C5 increases the risk of injury or death in animal models injected with aPL (16, 17). Our group has also investigated this relationship in a multicenter study (18). Utilizing pregnancy C3 and C4 normality ranges (in each trimester), we did not show an association between hypocomplementemia and obstetric complications in primary APS. Conversely, a prospective cohort study (19) that included both primary and secondary APS has recognized hypocomplementemia as an independent predictor of low birth weight and premature delivery.

As expected, T-APS group had the highest rate of complicated pregnancies (24.2%), confirming the previous reports (9). We did not find any statistical difference in the rate of APO among patients with or without a full blown picture of primary APS (O + T vs NC + carriers).

Till date, there are no generalized recommendations on how to treat women not fulfilling the APS criteria or if a prophylactic treatment is required during pregnancy and puerperium. A recent systematic review reported that LDA is comparable to the usual care or placebo in the prevention of pregnancy complications. However, the authors were able to include only a limited number of studies in their analysis (5). The majority of the patients included in that review belonged to a large retrospective observational Italian study on aPL patients that also included patients with organ or systemic autoimmune diseases (20). The authors concluded that, in their cohort, the most important factor related to pregnancy outcome was the antibody profile (medium–high titers of aPL) and not the treatment or the previous pregnancy morbidity. Hence, these results cannot be compared with those obtained in the present study in which all the patients received antithrombotic therapy. However, in the aPL carrier group, we observed a significant association between APO and triple aPL positivity, a feature included in the definition of the “high-risk” aPL profile (9).

Beside pregnancy morbidity, we reported the occurrence of a moderate number of thrombotic events during pregnancy or puerperium. The majority of the thrombotic events occurred during puerperium, despite the use of adequate antithrombotic treatments. This pattern is consistent to the well-known risk of postpartum thrombosis in the general obstetric population. The two cases of CAPS onset during the puerperium were already described in a retrospective series of 13 patients (8). In more than 90% of their cases (as well as in our two women), the occurrence of a HELLP syndrome can be considered as a predictive factor for CAPS.

This study has several limitations: the retrospective design, even if data were prospectively collected during each pregnancy; the lack of a centralized laboratory, although all the laboratories were referral centers; the wide temporal range of the pregnancies (15 years, 2000–2014); and the multicenter nature, possible source of heterogeneity. Nevertheless, APS is a rare disease, and a collaborative study including several pregnancy clinics was required in order to achieve a significant number of cases.

In conclusion, maternal and fetal complications were observed in nearly 18% of aPL-positive patients despite the conventional treatment according to the current recommendations. Additional immunomodulatory treatment might be required for these difficult patients (21, 22).

In the last two decades, a great improvement was certainly achieved in the outcome of APS pregnancies. This success is probably due to multi-specialistic teams devoted to the tight control of aPL-positive women. A preconception risk stratification is recognized as crucial. The results obtained in this study confirmed the role of some of the parameters lately included in the recently revised risk factors for APO in APS patients (23). In addition, we underline the influence of a nonsystemic autoimmune phenotype even in patients with aPL without a formal diagnosis of APS.

In the absence of controlled trials and with very limited prospective studies available, the present work, collecting a very large number of pregnancies from experienced centers, might contribute to a better definition of the clinical and laboratory features associated with a poor prognosis that deserve attention by the clinicians in the everyday practice.

## Ethics Statement

This study was performed according to the principles of the Declaration of Helsinki with written informed consent from all subjects and was approved by the Ethic Committee of the Promoting Centre (Brescia) (approval number 1088) and it has been approved by the other centers.

## Author Contributions

MF, LA, NC-C, and AT designed the study. MF, LA, ML, VL, NM, JP, AL, and SZ evaluated the patients. MF, EA, and EB recruited the patients. MF, LA, NC-C, and AT wrote the manuscript. All the coauthors reviewed the manuscript.

## Conflict of Interest Statement

The authors declare that the research was conducted in the absence of any commercial or financial relationships that could be construed as a potential conflict of interest.
